# *Lacticaseibacillus paracasei* KBL382 contributes to the immunomodulation in THP-1 cells

**DOI:** 10.71150/jm.2509016

**Published:** 2026-02-28

**Authors:** MinJoong Kim, Min Jung Jo, SungJun Park, Seoung Bum Lee, Sung Jae Jang, Cheonghoon Lee, Woon-Ki Kim, GwangPyo Ko

**Affiliations:** 1 Graduate School of Public Health, Seoul National University, Seoul 08826, Republic of Korea; 2 N-Bio, Seoul National University, Seoul 08826, Republic of Korea; 3 KoBioLabs, Inc., Seoul 08826, Republic of Korea; 4 weBiom Inc., Seoul 08826, Republic of Korea; 5 Institute of Health and Environment, Seoul National University, Seoul 08826, Republic of Korea; 6 Brain Korea for Global Leader of Better Environmental health (BK4GLOBE), Seoul National University, Seoul 08826, Republic of Korea

**Keywords:** immune response, *Lacticaseibacillus paracasei*, negative regulatory molecules, probiotic, Toll-like receptor signaling pathway

## Abstract

Gut microbiome imbalance can induce inflammatory responses via Toll-like receptor 2 (TLR2) signaling pathways. *Lactobacillus* spp., popularly applied as probiotics in both humans and animals, have come into the spotlight for their strong immunomodulatory effects. We aimed to evaluate the immunomodulatory potential of live or pasteurized *Lacticaseibacillus paracasei* (*L. paracasei*) KBL382, isolated from healthy Korean individuals, in an in vitro monocytic THP-1 cell model. Live *L. paracasei* KBL382 significantly increased TLR2 and MyD88 expressions and induced IRAK1 expression, irrespective of lipopolysaccharide (LPS) stimulation (*p* < 0.05). Under LPS stimulation, THP-1 cells treated with live *L. paracasei* KBL382 showed significantly increased interleukin (IL)-6 and IL-10 levels (*p* < 0.05). Pasteurized *L. paracasei* exhibited a decrease in IL-12 levels (*p* < 0.05). Moreover, live *L. paracasei* KBL382 also markedly elevated A20 and SOCS1 expressions, the critical negative regulators of inflammation, regardless of LPS stimulation (*p* < 0.05). The expression of IRAK3, another negative regulator of inflammation, was increased in THP-1 cells with live *L. paracasei* KBL382 under LPS stimulation (*p* < 0.05). Our findings demonstrate that *L. paracasei* KBL382 contributes to the immunomodulation in THP-1 cells by coordinating both positive and negative regulatory signaling. *L. paracasei* KBL382 could be used as a promising probiotic strain for attenuating chronic inflammation through the gut-immune axis mechanisms.

## Introduction

Live microorganisms bring out a range of health-promoting effects on their host (Hill et al., 2014). Especially, *Lactobacillus* spp., generally used as probiotics for human beings and animals, can restore immune imbalance due to allergic airway inflammation, lumbar disc herniation, and metabolic disorders (Lin et al., 2023; Wang et al., 2021; Zeng et al., 2021). *Lactiplantibacillus plantarum* (*L. plantarum*) Lp91 enhances the host gut barrier and modulates cytokine production, by decreasing tumor necrosis factor (TNF)-α and interleukin (IL)-6 (Sudhakaran et al., 2013). Additionally, cyclic peptides derived from lactic acid bacteria inhibit lipopolysaccharide (LPS)-triggered pro-inflammatory cytokine productions in activated immune cells (Saravanan et al., 2023). Several *Lactobacillus* strains also produce membrane vesicles with enhanced anti-inflammatory properties (Kim et al., 2020b).

In our previous studies, *Lacticaseibacillus paracasei* (*L. paracasei*) KBL382, isolated from healthy Korean individuals, showed robust potential in modulating irregular immune responses. *L. paracasei* KBL382 effectively attenuated symptoms of atopic dermatitis and colitis in mice (Kim et al., 2019, 2020a). Moreover, *L. paracasei* KBL382 facilitated the expansion of regulatory T cells and modulated mRNA expression in macrophages, influencing glycolysis and macrophage polarization (Han et al., 2020a; Kim et al., 2020a). These findings could support the development of innovative strategies for managing chronic inflammatory diseases through probiotic supplementation.

Gut microbiome imbalance can induce inflammatory responses through Toll-like receptor 2 (TLR2) signaling pathways. Specifically, lipoteichoic acid (LTA), a key structural element of the bacterial cell wall component, recognized by TLR2 and engages the myeloid differentiation primary response 88 (MyD88)-dependent pathway, facilitating downstream signaling cascades involving NF-κB and MAPK, which induces the production of pro-inflammatory cytokines, including IL-6 and TNF-α (Kawai et al., 2024; Pereira and Gazzinelli, 2023; Xia et al., 2021). However, recent studies have revealed that these pathways can be modulated by *Lacticaseibacillus rhamnosus* (*L. rhamnosus*) to reduce the excessive host immune responses (Duan et al., 2022; Pereira and Gazzinelli, 2023). Both live and pasteurized *Lactobacillus* strains exhibit strong immunomodulatory effects, as their bioactive components, including cell wall fragments and surface proteins, interact with the host immune system (Taverniti and Guglielmetti, 2011).

Therefore, we investigated the immunomodulatory effects of live or pasteurized *L. paracasei* KBL382 in an in vitro monocytic THP-1 cell model by assessing changes in biomarkers associated with the TLR2 signaling pathway, evaluating pro- or anti-inflammatory cytokine levels, and monitored key negative regulators of immune signaling, including A20, IRAK3, suppressor of cytokine signaling 1 (SOCS1), and Toll-interacting protein (TOLLIP). Together, these analyses provide a comprehensive understanding of the immunomodulatory roles of *L. paracasei* KBL382 in host cells.

## Materials and Methods

### Preparation of live and pasteurized *L. paracasei* KBL382

*L. paracasei* KBL382, isolated from healthy Korean individuals, was anaerobically cultured at 37°C for 24 h Lactobacilli MRS Agar (Becton, Dickinson and Company, USA) supplemented with 0.05% L-cysteine hydrochloride, as previously described (Han et al., 2020a). The collected bacterial cells were then washed twice with 1× PBS and pelleted by centrifugation at 1,200 × g at 4°C for 10 min. To prepare the inactivated cells, a total of 4 × 10^6^ CFUs of *L. paracasei* KBL382 was aliquoted per 3 ml 1× PBS in 15 ml conical tubes and subjected to a pasteurization process at 60°C for 30 min. No live bacterial cells were remained after the pasteurization process (data not shown). Both live and pasteurized bacteria were subsequently stored at 4°C until use (~1 day).

### Preparation of THP-1 cells

Monocytic THP-1 cells (ATCC TIB-202) were cultured in a 5% CO_2_ incubator at 37°C using RPMI 1640 medium (Thermo Fisher Scientific, USA) supplemented with 10% fetal bovine serum (FBS) and 1% antibiotic-antimycotic solution. The cells were differentiated into macrophages by subsequent incubation for 24 h at 37°C with 100 ng/ml phorbol 12-myristate 13-acetate (PMA; Sigma-Aldrich, USA) as described previously (Chanput et al., 2014; Starr et al., 2018).

### Measurement of mRNA expression in THP-1 cells

Approximately 2 × 10^5^ THP-1 cells were washed with 1× PBS and seeded into a 48-well plate (SPL Life Sciences, Korea). Then, cells were cultured in a 5% CO_2_ incubator at 37°C using RPMI 1640 medium supplemented with 10% FBS for 6 h and stimulated with 200 ng/ml LPS (Sigma-Aldrich) or left unstimulated for 24 h, as previously described with slight modification (Liu et al., 2016; Palsson-McDermott et al., 2015). To confirm the TLR2-related effects of *L. paracasei* KBL382, THP‑1 cells were separately seeded and treated with TLR2 inhibitor C29 (100 μM; HY-100461, MedChemExpress, USA) for 1 h. Subsequently, a total of 4 × 10^6^ CFUs of live or pasteurized *L. paracasei* KBL382 were added to the THP-1 or TLR2 inhibitor-treated THP-1 cells, while 1× PBS was used as a negative control.

Total RNA was extracted from the THP-1 cells using an easy-spin Total RNA Extraction Kit (iNtRON Biotechnology, Korea). Complementary DNA (cDNA) was then synthesized from the total RNA using a High-Capacity RNA-to-cDNA Kit (Thermo Fisher Scientific) according to the manufacturer’s instructions. Real-time quantitative PCR (qPCR) was subsequently performed on a StepOnePlus Real-Time PCR System (Thermo Fisher Scientific) with a Power SYBR Green PCR Master Mix (Thermo Fisher Scientific), and 0.01 mM primers (the final concentration for qPCR reaction: 0.5 µM) (Table 1). The qPCR reactions were conducted with an initial denaturation at 95°C for 5 min, followed by 40 cycles of 95°C for 5 s and 60°C for 10 s. All mRNA expression levels were normalized to glyceraldehyde-3-phosphate dehydrogenase (Han et al., 2020b).

### Measurement of TRAF6 in the protein level

After co-incubation of THP-1 cells with *L. paracasei* KBL382, the THP-1 cells were lysed with 1× RIPA buffer (Enzynomics, Korea) with the Halt protease inhibitor cocktail (Thermo Fisher Scientific). The cell lysate was centrifuged at 4°C for 15 min at 14,000 × g and the supernatant was collected. Protein concentration was measured using a BCA Protein Assay Kit (Thermo Fisher Scientific) according to the manufacturer’s instructions. Subsequently, 20 µg of the samples were run on a 4% to 15% gradient gel (Bio-Rad Laboratories, USA) at 100 V of constant voltage for 1 h. Separated proteins were transferred to a nitrocellulose membrane (GE Healthcare Life Science, Germany). The membrane was blocked with 5% Nonfat dry milk (Cell Signaling Technology, USA) for 1 h and incubated overnight at 4°C with rabbit anti-TRAF6 (#8028, Cell Signaling Technology) or rabbit anti-β-actin (#12620, Cell Signaling Technology). The visualization and quantification of bands was performed on a WSE-6200 LuminoGraph II System (ATTO, Japan) and CS Analyzer 4 (ATTO) with a NICSROWEST ECL Chemiluminescent kit (Bionics, Korea). All band intensities were normalized to the results of β-actin.

### Measurement of cytokine in the protein level

After co-incubation of THP-1 cells with *L. paracasei* KBL382, the cell culture supernatant was collected. The levels of various cytokines, including IL-6, IL-12, and IL-10 were measured using a BD CBA Human Inflammatory Cytokine Kit (BD Biosciences, USA), according to the manufacturer’s instructions.

### Statistical analysis

Data are presented as the Means ± the standard errors of the mean (SEMs) from three independent experiments. Statistical significance was determined using Kruskal-Wallis one-way analysis of variance (ANOVA) with Dunn’s post hoc test for multiple comparisons. A P-value (*p*) less than 0.05 was considered statistically significant. All statistical analyses and data visualization were performed using GraphPad Prism 10 (GraphPad Software, USA).

## Results

### Effects of *L. paracasei* KBL382 on TLRs and MyD88 expressions

**Fig. 1.**
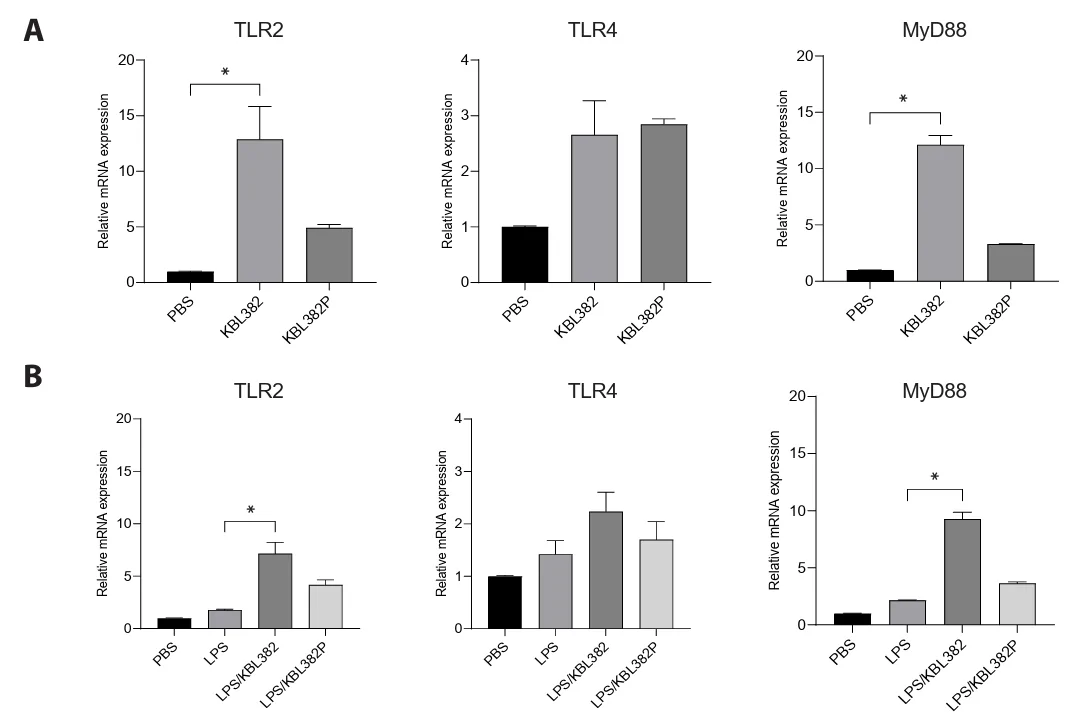
Effects of live and pasteurized *L. paracasei* KBL382 on TLRs and MyD88 expression in THP-1 cells. (A) In the absence of lipopolysaccharide (LPS) stimulation. (B) With LPS stimulation. Data are expressed as Means ± SEMs of three independent experiments. Asterisks indicate statistical significance (*p < 0.05; Kruskal-Wallis one-way ANOVA with Dunn’s post hoc test for multiple comparisons).

Live *L. paracasei* KBL382 significantly increased TLR2 and MyD88 expressions in THP-1 cells under LPS-free condition, compared to PBS-treated cells (*p* < 0.05) (Fig. 1A). Under LPS stimulation, TLR2 and MyD88 expressions were also increased in THP-1 cells with live *L. paracasei* KBL382, compared to LPS-treated cells (*p* < 0.05) (Fig. 1B). However, no significant changes in TLR4 expression were detected in THP-1 cells irrespective of LPS stimulation (Fig. 1A and 1B).

### Effects of *L. paracasei* KBL382 on TLR activation

**Fig. 2.**
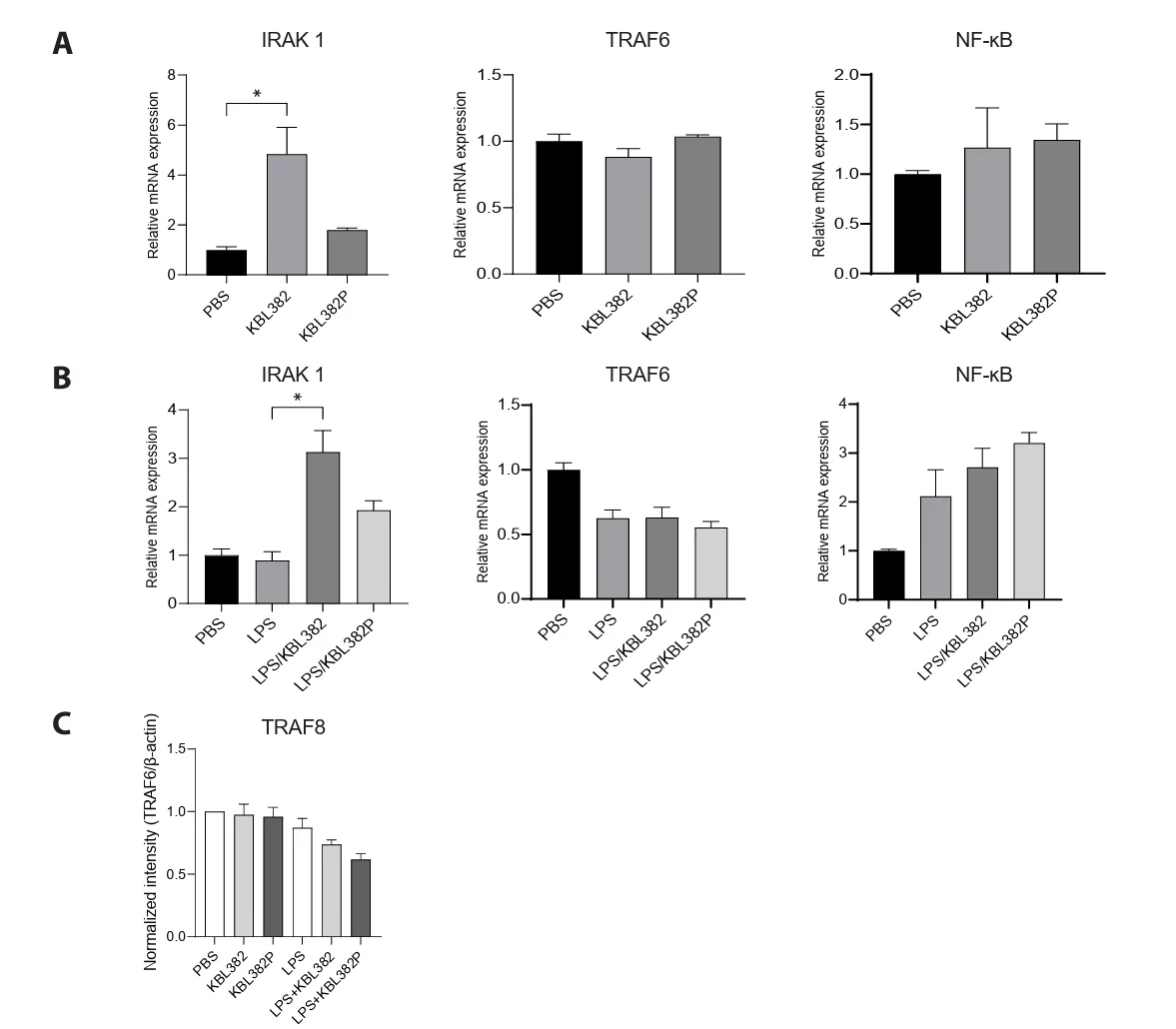
Effects of live and pasteurized *L. paracasei* KBL382 on biomarker expression related to TLR2 pathway signaling in THP-1 cells. (A) In the absence of LPS stimulation. (B) With LPS stimulation. (C) The changes in TRAF6 protein levels. Data are expressed as Means ± SEMs of three independent experiments. Asterisks indicate statistical significance (*p < 0.05; Kruskal-Wallis one-way ANOVA with Dunn’s post hoc test for multiple comparisons).

Live *L. paracasei* KBL382 treatment significantly induced IRAK1 expression, irrespective of LPS stimulation (*p* < 0.05) (Fig. 2A and 2B). However, both live and pasteurized *L. paracasei* KBL382-treated THP-1 cells did not exhibit significant increases in TRAF6 and NF-κB expressions with/without LPS stimulation (Fig. 2A–2C).

### Effects of *L. paracasei* KBL382 on cytokine levels

**Fig. 3.**
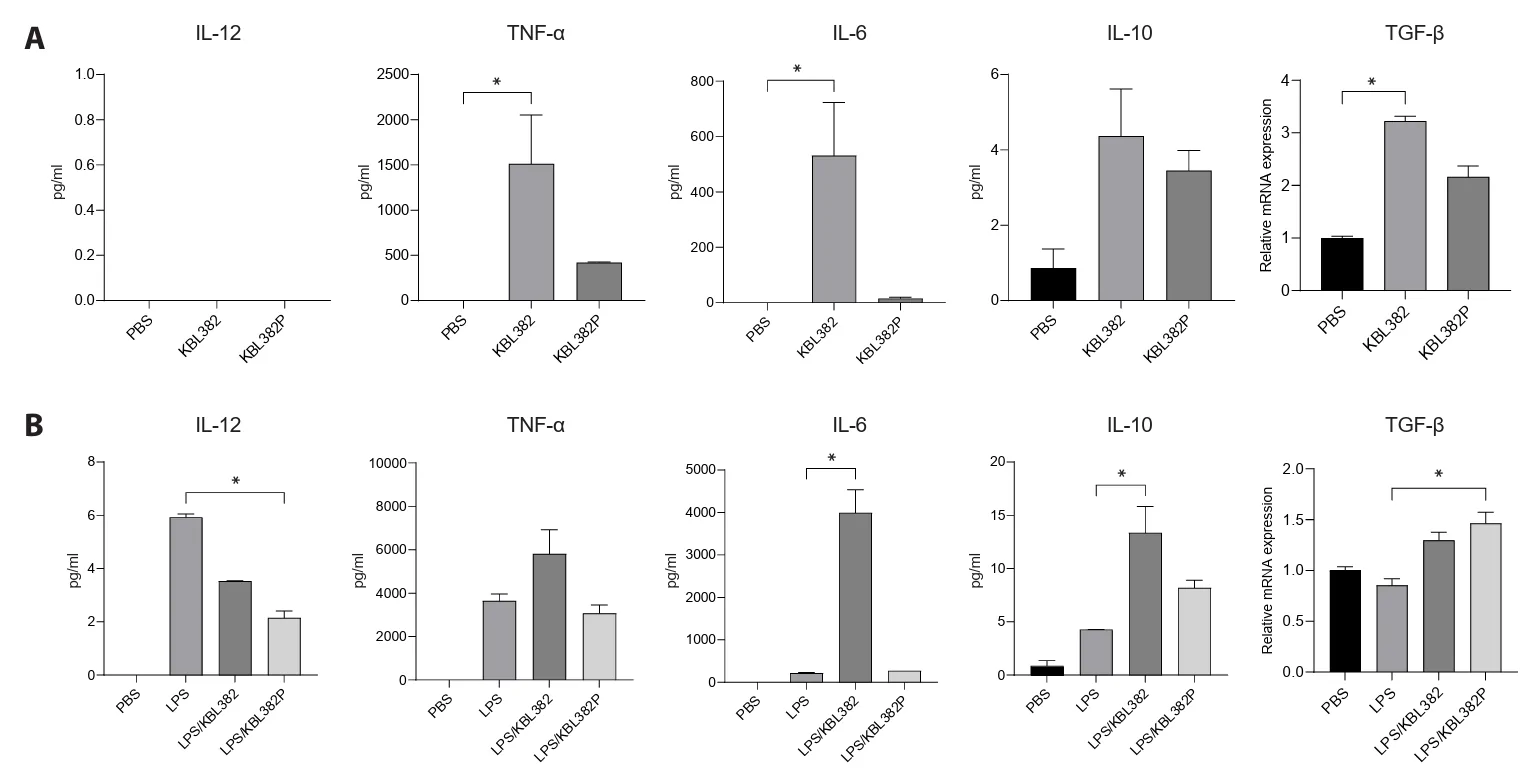
Effects of live and pasteurized *L. paracasei* KBL382 on cytokine production in THP-1 cells. (A) In the absence of LPS stimulation. (B) With LPS stimulation. Data are expressed as Means ± SEMs of three independent experiments. Asterisks indicate statistical significance (*p < 0.05; Kruskal-Wallis one-way ANOVA with Dunn’s post hoc test for multiple comparisons).

Under LPS-free condition, THP-1 cells with live *L. paracasei* KBL382 showed significant increases in IL-6, transforming growth factor (TGF)-β, and TNF-α levels, compared to PBS-treated cells (*p* < 0.05) (Fig. 3A). However, both live and pasteurized *L. paracasei* KBL382 did not affect the IL-12 level in THP-1 cells (Fig. 3A).

THP-1 cells with live *L. paracasei* KBL382 treatment also exhibited significant increases in IL-6 and IL-10 levels under LPS stimulation (*p* < 0.05) (Fig. 3B). Intriguingly, pasteurized *L. paracasei* KBL382 treated THP-1 cells showed the significant decrease in the IL-12 level and increase in TGF-β, compared to LPS-treated cells (*p* < 0.05) (Fig. 3B).

**Fig. 4.**
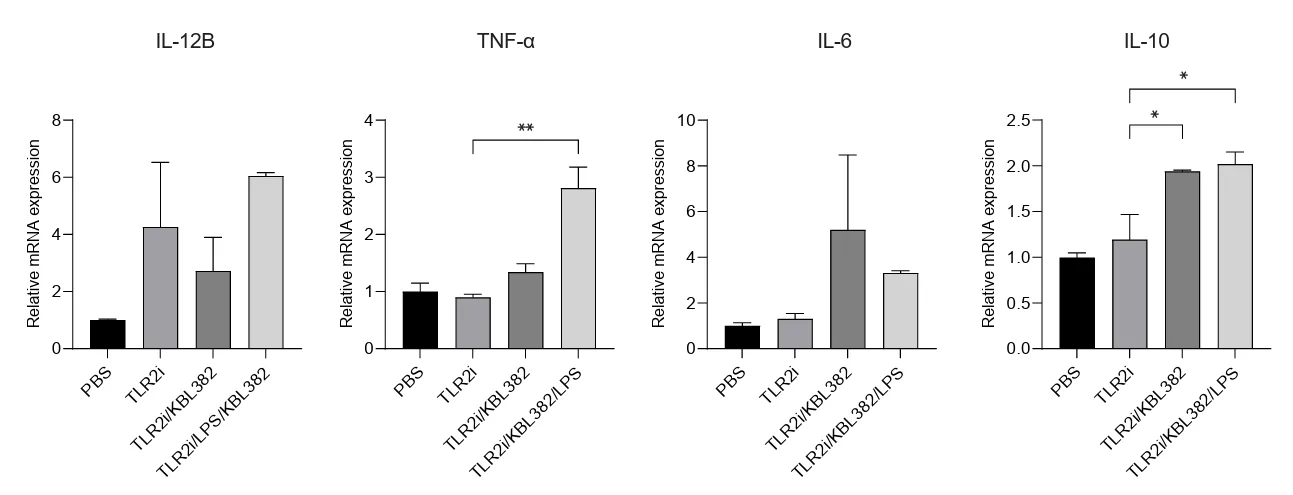
Effects of live *L. paracasei* KBL382 on cytokine expression in TLR-2 inhibitor treated THP-1 cells. Data are expressed as Means ± SEMs of three independent experiments. Asterisks indicate statistical significance (*p < 0.05, **p < 0.01; Kruskal-Wallis one-way ANOVA with Dunn’s post hoc test for multiple comparisons).

Treatment with live *L. paracasei* KBL382 significantly induced IL-10 expression in TLR2 inhibitor-treated THP-1 cells (*p* < 0.05) (Fig. 4). LPS-stimulated THP-1 cells with live *L. paracasei* KBL382 showed significant increase in TNF-α (*p* < 0.01) and IL-10 (*p* < 0.05) expression, regardless of TLR2 inhibitor treatment (Fig. 4).

### Effects of *L. paracasei* KBL382 on negative regulators of inflammation expressions

**Fig. 5.**
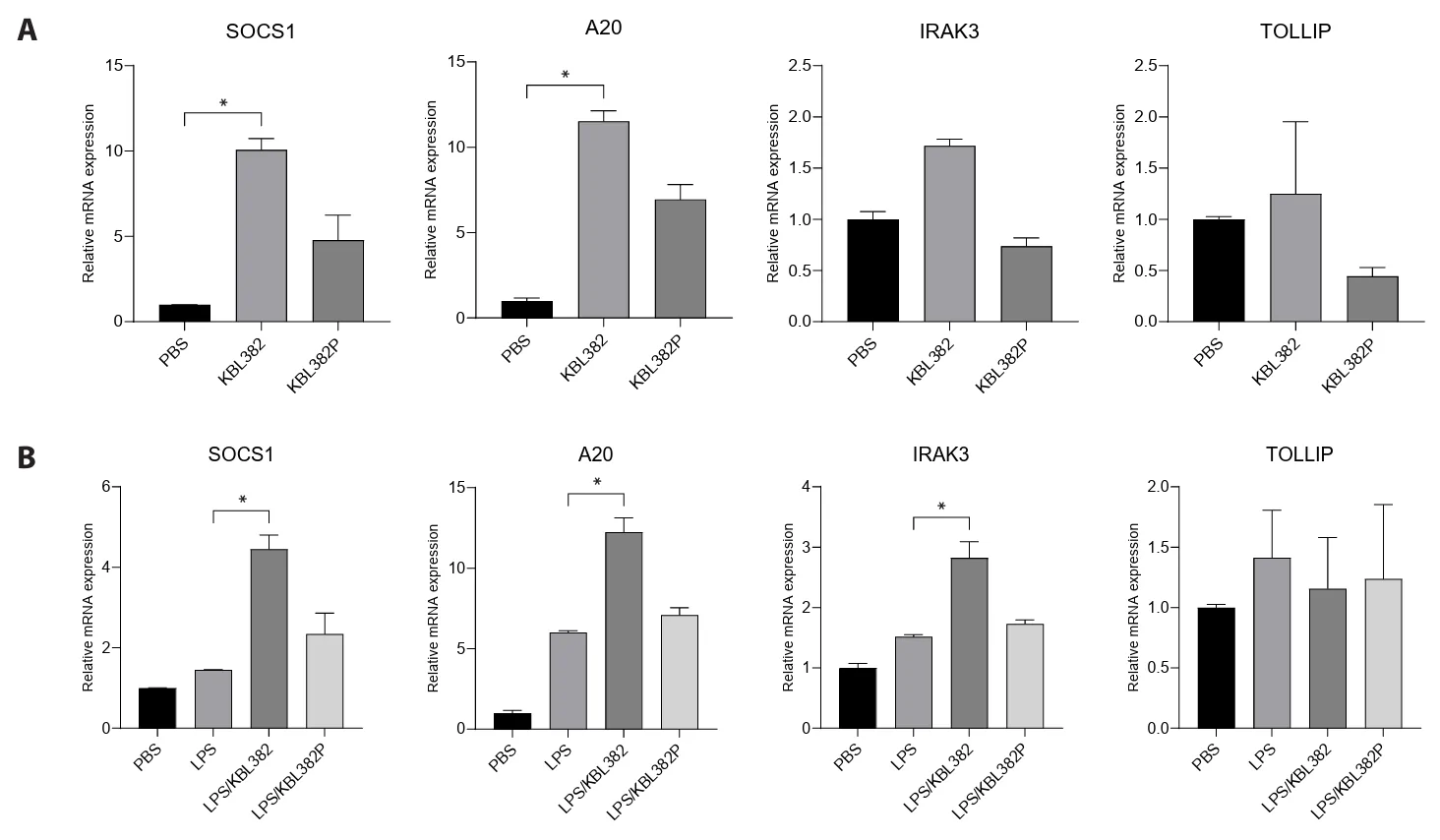
Effects of live and pasteurized *L. paracasei* KBL382 on the expression of negative regulators of inflammation in THP-1 cells. (A) In the absence of the LPS stimulation. (B) With LPS stimulation. Data are expressed as Means ± SEMs of three independent experiments. Asterisks indicate statistical significance (*p < 0.05; Kruskal-Wallis one-way ANOVA with Dunn’s post hoc test for multiple comparisons).

Live *L. paracasei* KBL382 treatment significantly increased A20 and SOCS1 expressions, regardless of LPS stimulation (*p* < 0.05) (Fig. 5A and 5B). Only live *L. paracasei* KBL382 treatment induced the significant increase in the IRAK3 expression under LPS stimulation compared to LPS-treated cells (*p* < 0.05) (Fig. 5B). No significant changes in TOLLIP expression were discovered in both live and pasteurized *L. paracasei* KBL382-treated THP-1 cells (Fig. 5A and 5B).

## Discussion

Our results suggest that *L. paracasei* KBL382 can modulate inflammatory responses in THP-1 cells through the TLR2 signaling pathway. Previous studies have demonstrated that several *Lactobacillus* strains effectively regulate immune-related diseases via interactions with TLRs. For example, *L. rhamnosus* GG prevents *Citrobacter rodentium*-induced colitis by activating TLR2-mediated anti-inflammatory pathways (Ryu et al., 2016). The *L. rhamnosus* GG extract mitigates osteoclast differentiation through downregulation of the TLR2/NF-κB signaling (Fu et al., 2024). Moreover, *Lactobacillus delbrueckii* CIDCA 133 attenuates mucositis due to chemotherapy and suppresses immune responses by modulating TLR2 and MyD88 signaling and reinforcing the epithelial barrier (Barroso et al., 2022). Especially, Live *L. paracasei* KBL382 did not significantly increase IL-6, IL-12B and TNF-α expression under TLR2 inhibitor-treated conditions, indicating that immunomodulation effects of *L. paracasei* KBL382 are highly dependent on the TLR2 signaling pathways rather than TLR4-related pathways (Fig. 4). Therefore, the administration of *L. paracasei* KBL382 may exert substantial host immunomodulatory effects.

In general, the effects of live *L. paracasei* KBL382 on the expression of TLRs and biomarkers associated with the TLR2 signaling pathway were much stronger than those of the pasteurized form in THP-1 cells (Figs. 1 and 2). Live probiotics retain intact microbial components and secrete various bioactive substances, that are the key contributors to host immune-related signaling systems (Bermudez-Brito et al., 2012). Therefore, even though pasteurized bacterial cells preserved some immunomodulatory capacity, their effects may have been reduced by heat treatment. Further investigation of the effector molecules of *L. paracasei* KBL382 and their structural changes due to heat treatment should be performed for elucidating the immunomodulatory mechanisms of *L. paracasei* KBL382.

Interestingly, live *L. paracasei* KBL382 treatment selectively increased IRAK1 expression in THP-1 cells and levels of IL-6 and TNF-α (Figs. 2 and 3A). IRAK1 can activate downstream signaling pathways, resulting in elevated pro-inflammatory cytokine levels (Wells, 2011; Xia et al., 2021). On the other hand, pasteurized *L. paracasei* KBL382 did not affect IL‑6 and TNF‑α levels but significantly reduced IL‑12 levels in THP‑1 cells under LPS stimulation (Fig. 3B). Pasteurized cell cannot produce its inherent metabolites and secrete proteins or other cellular components, therefore, the decrease in IL-12 due to *L. paracasei* KBL382 could be occurred by specific molecules located on its outer cellular components. Further studies employing multi-omics approaches are warranted to comprehensively elucidate the immunomodulatory mechanisms of *L. paracasei* KBL382.

Moreover, live *L. paracasei* KBL382 simultaneously increased the anti-inflammatory cytokine IL-10 and TGF-β (Fig. 3A and 3B). TGF-β can effectively suppress excessive responses of immune cells (Massagué and Sheppard, 2023) and the strong induction of TGF-β of various *Lactobacillus* spp., such as *Lactobacillus gasseri* SBT2055 and *L. plantarum* 22A-3 have been reported (Lamubol et al., 2021; Sakai et al., 2014). Pasteurized *L. paracasei* KBL382 also significantly induced TGF-β in LPS-stimulated THP-1 cells, indicating that outer cellular components of *L. paracasei* KBL382 could have a major role for controlling abnormal immune status via bi-directional approaches (Fig. 3B).

Figure 5 illustrates the induction of A20, IRAK3, and SOCS1, which are negative regulators of inflammation, that act as crucial brakes within TLR signaling pathways, preventing excessive inflammatory responses. A20 constrains NF-κB signaling, whereas IRAK3 dampens IRAK1 activation (Hubbard and Moore, 2010; Priem et al., 2020; Sobah et al., 2021; Thiel et al., 2023). SOCS1 suppresses both MyD88 and the MyD88-independent Toll/IL-1 receptor-domain-containing adapter-inducing interferon-β signaling cascade (Nakagawa et al., 2002). Several *Lactobacillus* strains, such as *Lactobacillus helveticus* SBT2171 and *Lactobacillus acidophilus*, can successfully promote A20 expression (Kawano et al., 2019; Li et al., 2016). The upregulation of A20, IRAK3, and SOCS1 gene expression in THP-1 cells indicates that the potential of modulate host immunity by inducing negative feedback mechanisms.

In conclusion, by orchestrating both positive and negative regulatory signaling, *L. paracasei* KBL382 treatment contributes to the immunomodulation in THP-1 cells. Our findings suggest that *L. paracasei* KBL382 can be developed as a promising tool for managing chronic inflammation through the gut-immune axis mechanisms. Further animal and human studies regarding the dose dependency of *L. paracasei* KBL382 could provide valuable insights into its immunomodulatory effects and safety.

## Figures and Tables

**Table 1.** Primers used in this study

Target	Sequence	Reference
A20	Fw: 5’- AACATTTTGCTGCTGCCTC -3’	Xiong et al. (2011)
	Rv: 5’- AGGTGCTTTGTGTGGTTCG -3’	
GAPDH	Fw: 5’- GGAAGGTGAAGGTCGGAGTC -3’	Han et al. (2020b)
	Rv: 5’- TCAGCCTTGACGGTGCCATG -3’	
IL-6	Fw: 5’- CATCCTCGACGGCATCTCAG -3’	Kanmani and Kim (2018)
	Rv: 5’- GCTCTGTTGCCTGGTCCTC -3’	
IL-10	Fw: 5’- TCAGGGTGGCGACTCTAT -3’	
	Rv: 5’- TGGGCTTCTTCTAAATCGTTC -3’	
IL-12B	Fw: 5’- GGCTCCATGAAGGTGCTAC -3’	Hor et al. (2014)
	Rv: 5’- GTTCAGCCTCAGAATGCAAAA -3’	
IRAK1	Fw: 5’- ACTGGCCCTTGGCAGCTC -3’	Rahemi et al. (2019)
	Rv: 5’- GGCCAGCTTCTGGACCATC -3’	
IRAK3	Fw: 5’- TGCAACGCGGGCAAA -3’	Sun et al. (2017)
	Rv: 5’- TTTAGTGATGTGGGAGGATCTTCA -3’	
MyD88	Fw: 5’- GAGCGTTTCGATGCTTCAT -3’	Zarember and Godowski (2002)
	Rv: 5’- CGGATCATCTCCTGCACAAA -3’	
NF-κB	Fw: 5’- TCAATGGCTACACAGGACCA -3’	Ren et al. (2019)
	Rv: 5’- CACTGTCACCTGGAAGCAGA -3’	
SOCS1	Fw: 5’- CTGGGATGCCGTGTTATTTT-3’	Lee et al. (2017)
	Rv: 5’- TAGGAGGTGCGAGTTCAGGT-3’	
TLR2	Fw: 5’- GCCAAAGTCTTGATTGATTGG-3’	Erdinest et al. (2014)
	Rv: 5’- TTGAAGTTCTCCAGCTCCTGG -3’	
TLR4	Fw: 5’- GGTGGAAGTTGAACGAATGG -3’	Asai et al. (2003)
	Rv: 5’- CCAGCAAGAAGCATCAGGTG -3’	
TNF-α	Fw: 5’- TCTCGAACCCCGAGTGACAA -3’	Ren et al. (2019)
	Rv: 5’- TATCTCTCAGCTCCACGCCA -3’	
TOLLIP	Fw: 5’-AGGTGACAACTGTCTCCGTC-3’	Sun et al. (2017)
	Rv: 5’-GCCAACTTTGCCTGTACCAC-3’	
TRAF6	Fw: 5’-CCTTTGGCAAATGTCATCTGTG-3’	Shen et al. (2013)
	Rv: 5’-CTCTGCATCTTTTCATGGCAAC-3’	
TGF-β	Fw: 5′-GAA GGC AGA GTT CAG GGT CTT-3′	Kwon et al. (2010)
	Rv: 5′-GGT TCC TGT CTT TGT GGT GAA-3′	

Fw: Represents a forward primer sequence. Rv: Represents a reverse primer sequence.

## Data Availability

All data supporting the findings of this study are available from the corresponding author upon reasonable request.

## References

[b1-jm-2509016] Asai Y, Jinno T, Ogawa T (2003). Oral treponemes and their outer membrane extracts activate human gingival epithelial cells through toll-like receptor 2. Infect Immun.

[b2-jm-2509016] Barroso FAL, de Jesus LCL, da Silva TF, Batista VL, Laguna J (2022). *Lactobacillus delbrueckii* CIDCA 133 ameliorates chemotherapy-induced mucositis by modulating epithelial barrier and TLR2/4/MyD88/NF-κB signaling pathway. Front Microbiol.

[b3-jm-2509016] Bermudez-Brito M, Plaza-Diaz J, Munoz-Quezada S, Gomez-Llorente C, Gil A (2012). Probiotic mechanisms of action. Ann Nutr Metab.

[b4-jm-2509016] Chanput W, Mes JJ, Wichers HJ (2014). THP-1 cell line: an in vitro cell model for immune modulation approach. Int Immunopharmacol.

[b5-jm-2509016] Duan T, Du Y, Xing C, Wang HY, Wang RF (2022). Toll-like receptor signaling and its role in cell-mediated immunity. Front Immunol.

[b6-jm-2509016] Erdinest N, Aviel G, Moallem E, Anteby I, Yahalom C (2014). Expression and activation of toll-like receptor 3 and toll-like receptor 4 on human corneal epithelial and conjunctival fibroblasts. J Inflamm.

[b7-jm-2509016] Fu J, Jia L, Wu L, Jiang Y, Zhao R (2024). *Lactobacillus rhamnosus* inhibits osteoclast differentiation by suppressing the TLR2/NF-κB pathway. Oral Dis.

[b8-jm-2509016] Han DH, Kim WK, Park S, Jang YJ, Ko G (2020a). *Lactobacillus paracasei* treatment modulates mRNA expression in macrophages. Biochem Biophys Rep.

[b9-jm-2509016] Han MH, Lee JH, Kim G, Lee E, Lee YR (2020b). Expression of the long noncoding RNA GAS5 correlates with liver fibrosis in patients with nonalcoholic fatty liver disease. Genes.

[b10-jm-2509016] Hill C, Guarner F, Reid G, Gibson GR, Merenstein DJ (2014). The International Scientific Association for Probiotics and Prebiotics consensus statement on the scope and appropriate use of the term probiotic. Nat Rev Gastroenterol Hepatol.

[b11-jm-2509016] Hubbard LL, Moore BB (2010). IRAK-M regulation and function in host defense and immune homeostasis. Infect Dis Rep.

[b12-jm-2509016] Hor YT, Voon DC, Koo JK, Wang H, Lau WM (2014). A role for RUNX3 in inflammation-induced expression of IL23A in gastric epithelial cells. Cell Rep.

[b13-jm-2509016] Kanmani P, Kim H (2018). Protective effects of lactic acid bacteria against TLR4-induced inflammatory response in hepatoma HepG2 cells through modulation of toll-like receptor negative regulators of mitogen-activated protein kinase and NF-κB signaling. Front Immunol.

[b14-jm-2509016] Kawai T, Ikegawa M, Ori D, Akira S (2024). Decoding toll-like receptors: recent insights and perspectives in innate immunity. Immunity.

[b15-jm-2509016] Kawano M, Miyoshi M, Miyazaki T (2019). *Lactobacillus helveticus* SBT2171 induces A20 expression via toll-like receptor 2 signaling and inhibits the lipopolysaccharide-induced activation of nuclear factor-kappa B and mitogen-activated protein kinases in peritoneal macrophages. Front Immunol.

[b16-jm-2509016] Kim WK, Jang YJ, Han DH, Jeon K, Lee C (2020a). *Lactobacillus paracasei* KBL382 administration attenuates atopic dermatitis by modulating immune response and gut microbiota. Gut Microbes.

[b17-jm-2509016] Kim WK, Jang YJ, Seo B, Han DH, Park S (2019). Administration of *Lactobacillus paracasei* strains improves immunomodulation and changes the composition of gut microbiota leading to improvement of colitis in mice. J Funct Foods.

[b18-jm-2509016] Kim W, Lee EJ, Bae IH, Myoung K, Kim ST (2020b). *Lactobacillus* plantarum-derived extracellular vesicles induce anti-inflammatory M2 macrophage polarization in vitro. J Extracell Vesicles.

[b19-jm-2509016] Kwon HK, Lee CG, So JS, Chae CS, Hwang JS (2010). Generation of regulatory dendritic cells and CD4^+^Foxp3^+^ T cells by probiotics administration suppresses immune disorders. Proc Natl Acad Sci USA.

[b20-jm-2509016] Lamubol J, Ohto N, Kuwahara H, Mizuno M (2021). *Lactiplantibacillus plantarum* 22A-3-induced TGF-β1 secretion from intestinal epithelial cells stimulates CD103^+^ DC and Foxp3^+^ Treg differentiation and ameliorates colitis in mice. Food Funct.

[b21-jm-2509016] Lee SW, Liu CW, Hu JY, Chiang LM, Chuu CP (2017). Suppressors of cytokine signaling in tuberculosis. PLoS One.

[b22-jm-2509016] Li H, Zhang L, Chen L, Zhu Q, Wang W (2016). *Lactobacillus acidophilus* alleviates the inflammatory response to enterotoxigenic *Escherichia coli* K88 via inhibition of the NF-κB and p38 mitogen-activated protein kinase signaling pathways in piglets. BMC Microbiol.

[b23-jm-2509016] Lin EK, Chang WW, Jhong JH, Tsai WH, Chou CH (2023). *Lacticaseibacillus paracasei* GM-080 ameliorates allergic airway inflammation in children with allergic rhinitis: from an animal model to a double-blind, randomized, placebo-controlled trial. Cells.

[b24-jm-2509016] Liu L, Lu Y, Martinez J, Bi Y, Lian G (2016). Proinflammatory signal suppresses proliferation and shifts macrophage metabolism from Myc-dependent to HIF1α-dependent. Proc Natl Acad Sci USA.

[b25-jm-2509016] Massagué J, Sheppard D (2023). TGF-β signaling in health and disease. Cell.

[b26-jm-2509016] Nakagawa R, Naka T, Tsutsui H, Fujimoto M, Kimura A (2002). SOCS-1 participates in negative regulation of LPS responses. Immunity.

[b27-jm-2509016] Palsson-McDermott EM, Curtis AM, Goel G, Lauterbach MA, Sheedy FJ (2015). Pyruvate kinase M2 regulates HIF-1α activity and IL-1β induction and is a critical determinant of the Warburg effect in LPS-activated macrophages. Cell Metab.

[b28-jm-2509016] Pereira M, Gazzinelli RT (2023). Regulation of innate immune signaling by IRAK proteins. Front Immunol.

[b29-jm-2509016] Priem D, van Loo G, Bertrand MJM (2020). A20 and cell death-driven inflammation. Trends Immunol.

[b30-jm-2509016] Rahemi S, Nematollahi-Mahani SN, Rajaie A, Fallah H (2019). Inhibitor of interleukin-1 receptor-associated kinases 1/4, can increase the sensitivity of breast cancer cells to methotrexate. Int J Mol Cell Med.

[b31-jm-2509016] Ren D, Wang D, Liu H, Shen M, Yu H (2019). Two strains of probiotic *Lactobacillus* enhance immune response and promote naive T cell polarization to Th1. Food Agric Immunol.

[b32-jm-2509016] Ryu SH, Park JH, Choi SY, Jeon HY, Park JI (2016). The probiotic *Lactobacillus* prevents *Citrobacter rodentium*-induced murine colitis in a TLR2-dependent manner. J Microbiol Biotechnol.

[b33-jm-2509016] Sakai F, Hosoya T, Ono-Ohmachi A, Ukibe K, Ogawa A (2014). *Lactobacillus gasseri* SBT2055 induces TGF-β expression in dendritic cells and activates TLR2 signaling to produce IgA in the small intestine. PLoS One.

[b34-jm-2509016] Saravanan P, R P, Balachander N, K KRS, S S et al (2023). Anti-inflammatory and wound healing properties of lactic acid bacteria and its peptides. Folia Microbiol.

[b35-jm-2509016] Shen J, Qiao Y, Ran Z, Wang T (2013). Differential activation of TRAF4 and TRAF6 in inflammatory bowel disease. Mediators Inflamm.

[b36-jm-2509016] Sobah ML, Liongue C, Ward AC (2021). SOCS proteins in immunity, inflammatory diseases, and immune-related cancer. Front Med.

[b37-jm-2509016] Starr T, Bauler TJ, Malik-Kale P, Steele-Mortimer O (2018). The phorbol 12-myristate-13-acetate differentiation protocol is critical to the interaction of THP-1 macrophages with *Salmonella* Typhimurium. PLoS One.

[b38-jm-2509016] Sudhakaran AV, Panwar H, Chauhan R, Duary RK, Rathore RK (2013). Modulation of anti-inflammatory response in lipopolysaccharide-stimulated human THP-1 cell line and mouse model at gene expression level with indigenous putative probiotic *lactobacilli*. Genes Nutr.

[b39-jm-2509016] Sun KY, Xu DH, Xie C, Plummer S, Tang M (2017). *Lactobacillus paracasei* modulates LPS-induced inflammatory cytokine release by monocyte-macrophages via up-regulation of negative regulators of NF-kappaB signaling in a TLR2-dependent manner. Cytokine.

[b40-jm-2509016] Taverniti V, Guglielmetti S (2011). The immunomodulatory properties of probiotic microorganisms beyond their viability (ghost probiotics: proposal of paraprobiotic concept). Genes Nutr.

[b41-jm-2509016] Thiel FG, Asgarbeik S, Glaubitz J, Wilden A, Lerch MM (2023). IRAK3-mediated suppression of pro-inflammatory MyD88/IRAK signaling affects disease severity in acute pancreatitis. Sci Rep.

[b42-jm-2509016] Wang Z, Wu H, Chen Y, Che H, Wang X (2021). *Lactobacillus paracasei* S16 alleviates lumbar disc herniation by modulating inflammatory response and gut microbiota. Front Nutr.

[b43-jm-2509016] Wells JM (2011). Immunomodulatory mechanisms of lactobacilli. Microb Cell Fact.

[b44-jm-2509016] Xia P, Wu Y, Lian S, Yan L, Meng X (2021). Research progress on toll-like receptor signal transduction and its roles in antimicrobial immune responses. Appl Microbiol Biotechnol.

[b45-jm-2509016] Xiong Y, Qiu F, Piao W, Song C, Wahl LM (2011). Endotoxin tolerance impairs IL-1 receptor-associated kinase 4 and TGF-β-activated kinase 1 activation, K63-linked polyubiquitination and assembly of IRAK1, TNF receptor-associated factor 6, and IκB kinaseγ and increases A20 expression. J Biol Chem.

[b46-jm-2509016] Zarember KA, Godowski PJ (2002). Tissue expression of human toll-like receptors and differential regulation of toll-like receptor mRNAs in leukocytes in response to microbes, their products, and cytokines. J Immunol.

[b47-jm-2509016] Zeng Z, Guo X, Zhang J, Yuan Q, Chen S (2021). *Lactobacillus paracasei* modulates the gut microbiota and improves inflammation in type 2 diabetic rats. Food Funct.

